# Effects on the QT Interval of a Gatifloxacin-Containing Regimen versus Standard Treatment of Pulmonary Tuberculosis

**DOI:** 10.1128/AAC.01834-16

**Published:** 2017-06-27

**Authors:** Piero L. Olliaro, Corinne Merle, Thuli Mthiyane, Boubacar Bah, Ferdinand Kassa, Evans Amukoye, Alimatou N′Diaye, Christian Perronne, Christian Lienhardt, Helen McIlleron, Katherine Fielding

**Affiliations:** a Special Programme on Research and Training in Tropical Disease (WHO/TDR), Geneva, Switzerland; b London School of Hygiene and Tropical Medicine, London, United Kingdom; c Medical Research Council, Durban, South Africa; d Service de Pneumo-phtisiologie, Hôpital Ignace Deen, Conakry, Guinea; e Centre National Hospitalier de Pneumo-phtisiologie, Cotonou, Benin; f Kenya Medical Research Institute, Nairobi, Kenya; g Programme National de Lutte contre la Tuberculose, Dakar, Senegal; h Hopitaux Universitaires Paris Ile-de-France Ouest, Assistance Publique-Hopitaux de Paris, Paris, Paris, France; i Institut de Recherche pour le Développement, Marseille, France; j Global Programme for Tuberculosis, World Health Organization, Geneva, Switzerland; k Division of Clinical Pharmacology, Department of Medicine, University of Cape Town, Cape Town, South Africa

**Keywords:** gatifloxacin, Mycobacterium tuberculosis, QT interval, cardiotoxicity, fluoroquinolones

## Abstract

The effects on ventricular repolarization—recorded on the electrocardiogram (ECG) as lengthening of the QT interval—of acute tuberculosis and those of standard and alternative antituberculosis regimens are underdocumented. A correction factor (QTc) is introduced to make the QT independent of the heart rate, translating into the slope of the regression line between QT and heart rate being close to zero. ECGs were performed predosing and 1 to 5 h postdosing (month 1, month 2, and end of treatment) around drugs' peak concentration time in tuberculosis patients treated with either the standard 6-month treatment (rifampin and isoniazid for 6 months and pyrazinamide and ethambutol for 2 months; “control”) or a test regimen with gatifloxacin, rifampin, and isoniazid given for 4 months (pyrazinamide for the first 2 months) as part of the OFLOTUB study, a randomized controlled trial conducted in five African countries. Drug levels were measured at steady state (month 1) in a subset of patients. We compared treatment effects on the QTc and modeled the effect of individual drugs' maximum concentrations of drug in serum (*C*_max_) on the Fridericia-corrected QT interval. A total of 1,686 patients were eligible for the correction factor analysis of QT at baseline (mean age, 30.7 years; 27% female). Median heart rate decreased from 96/min at baseline to 71/min at end of treatment, and body temperature decreased from 37.2 to 36.5°C. Pretreatment, the nonlinear model estimated the best correction factor at 0.4081 in between Bazett's (0.5) and Fridericia's (0.33) corrections. On treatment, Fridericia (QTcF) was the best correction factor. A total of 1,602 patients contributed to the analysis of QTcF by treatment arm. The peak QTcF value during follow-up was >480 ms for 21 patients (7 and 14 in the test and control arms, respectively) and >500 ms for 9 patients (5 and 4, respectively), corresponding to a risk difference of −0.9% (95% confidence interval [CI], −2.0% to 2.3%; *P* = 0.12) and 0.1% (95% CI, −0.6% to 0.9%; *P* = 0.75), respectively, between the test and control arms. One hundred six (6.6%) patients had a peak measurement change from baseline of >60 ms (adjusted between-arm difference, 0.8%; 95% CI, −1.4% to 3.1%; *P* = 0.47). No evidence was found of an association between *C*_max_ of the antituberculosis drugs 1 month into treatment and the length of QTcF. Neither a standard 6-month nor a 4-month gatifloxacin-based regimen appears to carry a sizable risk of QT prolongation in patients with newly diagnosed pulmonary tuberculosis. This is to date the largest data set studying the effects of antituberculosis regimens on the QT, both for the standard regimen and for a fluoroquinolone-containing regimen. (This study has been registered at ClinicalTrials.gov under identifier NCT00216385.)

## INTRODUCTION

The time for ventricular depolarization and repolarization is measured on the surface electrocardiogram (ECG) as the time from the start of the Q wave to the end of the T wave. Prolonged repolarization is recorded on ECG as lengthening of the QT interval (1). This condition is considered to increase the risk for ventricular arrhythmias and the potentially fatal “torsades de pointes” (TdP). Ventricular repolarization is mediated mostly by the outflow of potassium (K^+^) from the myocytes. Attenuation of the voltage-dependent K^+^ channels' ability to repolarize can prolong the QT interval and create the conditions for TdP.

There is very little knowledge about how acute tuberculosis (TB) affects the QT interval or about the potential for antituberculosis treatments to affect ventricular repolarization. With prospects of having them added to the antituberculosis armory of drugs, drugs belonging to the fluoroquinolone (FQ) family have attracted attention, as they can variably affect ventricular repolarization (2). These drugs have different affinities for binding to the rapid component of the delayed-rectifier current I_Kr_, which is expressed by the human ether-a-go-go-related gene (hERG) (3). In particular, it has been suggested that compounds such as gatifloxacin and moxifloxacin, both considered in antituberculosis regimens, which have a methoxy substitution at position C-8, might inhibit hERG at therapeutically achievable concentrations (4).

Establishing the risk for QT prolongation associated with the use of a drug is not straightforward. The length of the QT interval varies during the day and from day to day and with gender and age and is influenced by potassium levels, body temperature, heart rate (HR), and factors such as disease and drugs. It is customary to introduce a correction factor to account for the effect of the heart rate (heart rate-corrected QT [QTc]). The correction factor is introduced to make the QT independent of the heart rate, hence the need for the slope of the regression line to be as close to zero as possible when the QT is plotted against the heart rate. The QTc is calculated by dividing the QT by RR (calculated as 60/heart rate). The International Conference for Harmonization (ICH) recommends analyzing the QT using the Bazett and the Fridericia corrections (QTcB and QTcF, respectively), which use fixed exponents of 0.5 and 0.33, respectively, for the RR, and exploring other corrections whenever appropriate. The Bazett correction QTcB (QT/RR^0.5^) is considered most suited for HRs of 60 to 100 beats per min (bpm) (it undercorrects if HR is <60 and overcorrects if HR is >100 bpm); the Fridericia formula QTcF (QT/RR^0.33^) is generally regarded as more appropriate outside this range. Various other corrections exist. Population-based corrections are also recommended for specific conditions (5, 6). There is no information on the appropriateness of these corrections in patients with pulmonary tuberculosis (PTB), i.e., how good they are in making the QT interval independent of the heart rate.

We analyzed the QT of patients with PTB enrolled in a randomized trial with a noninferiority design comparing the standard 6-month treatment to a gatifloxacin-containing 4-month regimen (the OFLOTUB trial [ClinicalTrials.gov registration no. NCT00216385]) (7). We also evaluated the effect of exposure, expressed as maximum concentration of drug in serum (*C*_max_) of the individual drugs of both treatment arms, in the patients who participated in a pharmacokinetic substudy (nested pharmacokinetic/pharmacodynamic [PK/PD] study).

## RESULTS

### Patients' characteristics.

**FIG 1:**
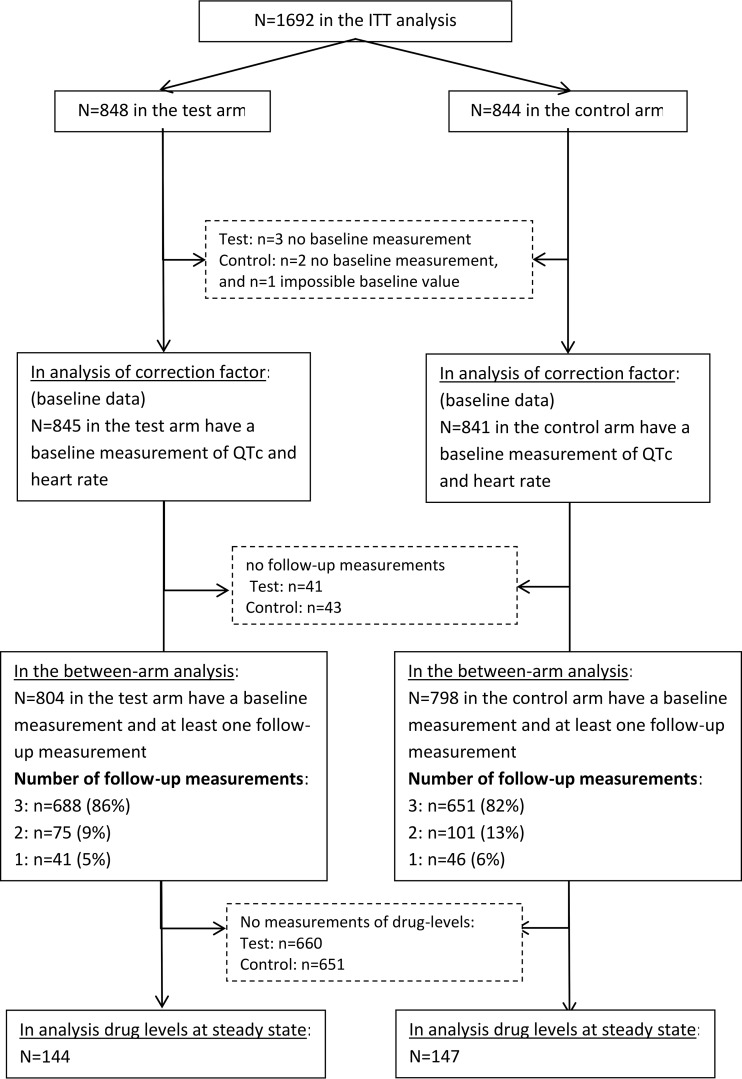
Study flow diagram.

Of the 1,692 patients in the intention-to-treat (ITT) population, 1,686 (99.6%) were eligible for the correction factor analysis of QT at baseline (flow diagram in Fig. 1). Mean age was 30.7 years, 27% were female, 18% were HIV positive, 51% had cavitation, and 25% had a temperature of >37.7°C (Table 1).

**TABLE 1:** Baseline demographics and clinical variables for patients in the correction factor analysis (*n* = 1,686) and the comparison of QTc by treatment arm (*n* = 1,602)

Demographic characteristic or clinical variable	Correction factor analysis (*n* = 1,686)		Between-arm comparison (*n* = 1,602)	
	Test (*n* = 845)	Control (*n* = 841)	Test (*n* = 804)	Control (*n* = 798)
No. of patients from country (%)				
Benin	158 (18.7)	158 (18.8)	150 (18.7)	151 (18.9)
Guinea	219 (25.9)	225 (26.7)	216 (26.9)	213 (26.7)
Kenya	100 (11.8)	97 (11.5)	100 (12.4)	95 (11.9)
Senegal	178 (21.1)	180 (21.4)	154 (19.1)	163 (20.4)
South Africa	190 (22.5)	181 (21.5)	184 (22.9)	176 (22.1)
Mean age, yr (SD)	30.8^*a*^ (9.1)	30.6 (9.0)	30.8 (9.1)	30.6 (8.9)
No. female (%)	229 (27.1)	232 (27.6)	215 (26.7)	224 (28.1)
No. HIV positive (%)^*b*^	147 (17.5)	156 (18.8)	141 (17.6)	150 (18.9)
No. with cavitation (%)^*c*^	438 (52.0)	417 (50.0)	413 (51.5)	394 (49.8)
Mean heart rate (SD)^*d*^	95.6 (17.4)	95.1 (17.8)	95.6 (17.5)	95.2 (17.5)
No. with temp >37.7°C (%)^*e*^	216 (25.6)	198 (23.6)	201 (25.0)	187 (23.5)
Mean BMI (SD)^*f*^	17.4 (4.9)	17.5 (5.0)	17.3 (4.9)	17.5 (5.0)

a Age not known for *n* = 1 in the test arm.

b HIV status unknown in the correction factor analysis for *n* = 11 (*n* = 5 in the test arm, *n* = 6 in the control arm) and in the between-arm comparison for *n* = 10 (*n* = 5 in the test arm, *n* = 5 in the control arm).

c Cavitary status unknown in the correction factor analysis for *n* = 10 (*n* = 3 in the test arm, *n* = 7 in the control arm) and in the between-arm comparison for *n* = 9 (*n* = 2 in the test arm, *n* = 7 in the control arm).

d Heart rate unknown in the between-arm comparison for *n* = 4 (*n* = 2 in the test arm, *n* = 2 in the control arm).

e Temperature unknown in the correction factor analysis for *n* = 4 (*n* = 1 in the test arm, *n* = 3 in the control arm) and in the between-arm comparison for *n* = 3 (*n* = 1 in the test arm, *n* = 2 in the control arm).

f BMI, body mass index.

### Heart rate and temperature.

Median heart rate decreased progressively from 96/min at baseline throughout treatment to reach 71 at the end of treatment. Median baseline body temperature was 37.2°C and decreased to approximately 36.5°C on treatment. The percentage of participants with temperatures of >37.7°C fell over follow-up to 2.7% (43/1,582) and 2.4% (37/1,539) at months 1 and 2 after the start of TB treatment, respectively, and to 0.7% (10/1,445) at the end of treatment (Table 2).

**TABLE 2:** Heart rate and temperature during the treatment phase, restricted to samples with data available for the correction analysis

Sampling time	Heart rate			Temp (°C)			% (no./total no.) of patients with temp of >37. 7°C
	Median	IQR^*b*^	*n*	Median	IQR	*n*	
Baseline	96	83–106	1,686	37.2	36.6–37.7	1,682	24.6 (414/1,682)
Month 1	81	71–95	1,562	36.6	36–37	1,582	2.7 (43/1,582)
Month 2	78	68–90	1,512	36.5	36–36.9	1,512	2.4 (37/1,539)
End of treatment^*a*^	71	62–81	1,402	36.4	36–36.9	1,445	0.7 (10/1,445)

a Month 4 (gatifloxacin) or month 6 (control).

b IQR, interquartile range.

### Correction factors.

In these patients with active PTB about to initiate treatment, the uncorrected QT increased with the heart rate overall (coefficient −202.7; 95% confidence interval [CI], −209.6 to −195.9; adjusted *R*^2^ = 0.67) (Table 3).

**TABLE 3:** Linear regression coefficients (gradient and intercept) for uncorrected QT and Bazett (QTcB), Fridericia (QTcF), and new (QTcTB) corrections versus 1-RR at baseline (randomization), 1 and 2 months from start of treatment, and end of treatment

QT and parameter	Value at sampling time (*n*):			
	Baseline (*n* = 1,686)	Month 1 (*n* = 1,560)	Month 2 (*n* = 1,512)	End of treatment^*a*^ (*n* = 1,402)
Uncorrected QT				
Gradient (95% CI)	−202.7 (−209.6, −195.9)	−162.3 (−168.8, −155.8)	−154.5 (−161.1, −148.0)	−134.4 (−141.3, −126.7)
Intercept (95% CI)	403.8 (401.2, 406.3)	396.3 (394.4, 398.3)	395.1 (393.4, 396.9)	395.1 (391.9, 395.0)
*R*^2^	0.67	0.61	0.59	0.48
QTcB				
Gradient (95% CI)	51.8 (43.5, 60.1)	80.8 (73.5, 88.1)	80.8 (73.6, 88.0)	90.0 (82.3, 97.7)
Intercept (95% CI)	397.1 (394.1, 400.2)	394.1 (392.0, 396.3)	394.5 (392.5, 396.4)	394.7 (393.0, 396.3)
*R*^2^	0.08	0.23	0.24	0.27
QTcF				
Gradient (95% CI)	−46.3 (−54.1, −38.5)	−9.8 (−16.8, −2.8)	−5.5 (−12.5, 1.4)	9.7 (2.2, 17.2)
Intercept (95% CI)	401.0 (398.1, 403.9)	395.4 (393.3, 397.4)	394.9 (392.0, 396.7)	394.1 (392.5, 395.7)
*R*^2^	0.075	0.005	0.002	0.005
QTcTB^*b*^				
Gradient (95% CI)	−2.9 (−10.9, 5.1)	30.7 (23.6, 37.8)	33.3 (26.2, 40.3)	46.0 (38.4, 53.6)
Intercept (95% CI)	399.5 (396.5, 402.4)	394.9 (392.8, 397.0)	394.7 (392.8, 396.6)	394.3 (392.7, 395.9)
*R*^2^	0.000	0.044	0.053	0.092
QTcB, % (no.)				
>450	5.5 (93)	7.0 (109)	6.7 (101)	6.3 (88)
>480	0.3 (5)	1.22 (19)	1.2 (18)	1.1 (16)
>500	0.1 (2)	0.7 (11)	0.5 (8)	0.2 (3)
QTcF, % (no.)				
>450	0.24 (4)	1.7 (27)	1.3 (20)	2.2 (31)
>480	0.06 (1)	0.5 (8)	0.3 (5)	0.6 (8)
>500	0 (0)	0.1 (2)	0.2 (3)	0.3 (4)
QTcTB,^*b*^ % (no.)				
>450	0.89 (15)	2.4 (38)	2.7 (41)	3.3 (46)
>480	0.12 (2)	0.6 (10)	0.5 (8)	0.78 (11)
>500	0.06 (1)	0.51 (8)	0.2 (3)	0.29 (4)

a Month 4 (gatifloxacin) or month 6 (control).

b Correction factor, 0.4081 (95% CI, 0.3949, 0.4213).

**FIG 2:**
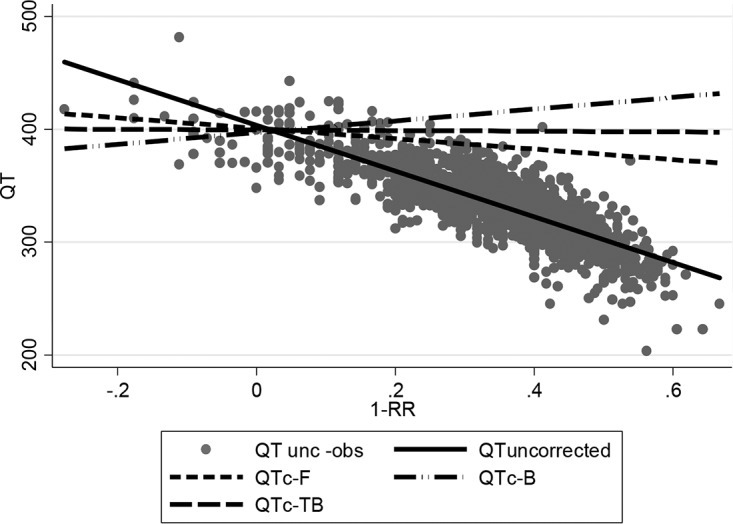
Plot of uncorrected data and regression line and regression lines for Bazett-corrected, Fridericia-corrected, and new-corrected QT (QTc-TB), using data at baseline (*n* = 1,686). QT unc-obs, QT uncorrected observed data; QT uncorrected, uncorrected regression line; QTc-F, Fridericia-corrected regression line; QTc-B, Bazett-corrected regression line; QTc-TB, regression line corrected using correction factor of 0.4081.

**FIG 3:**
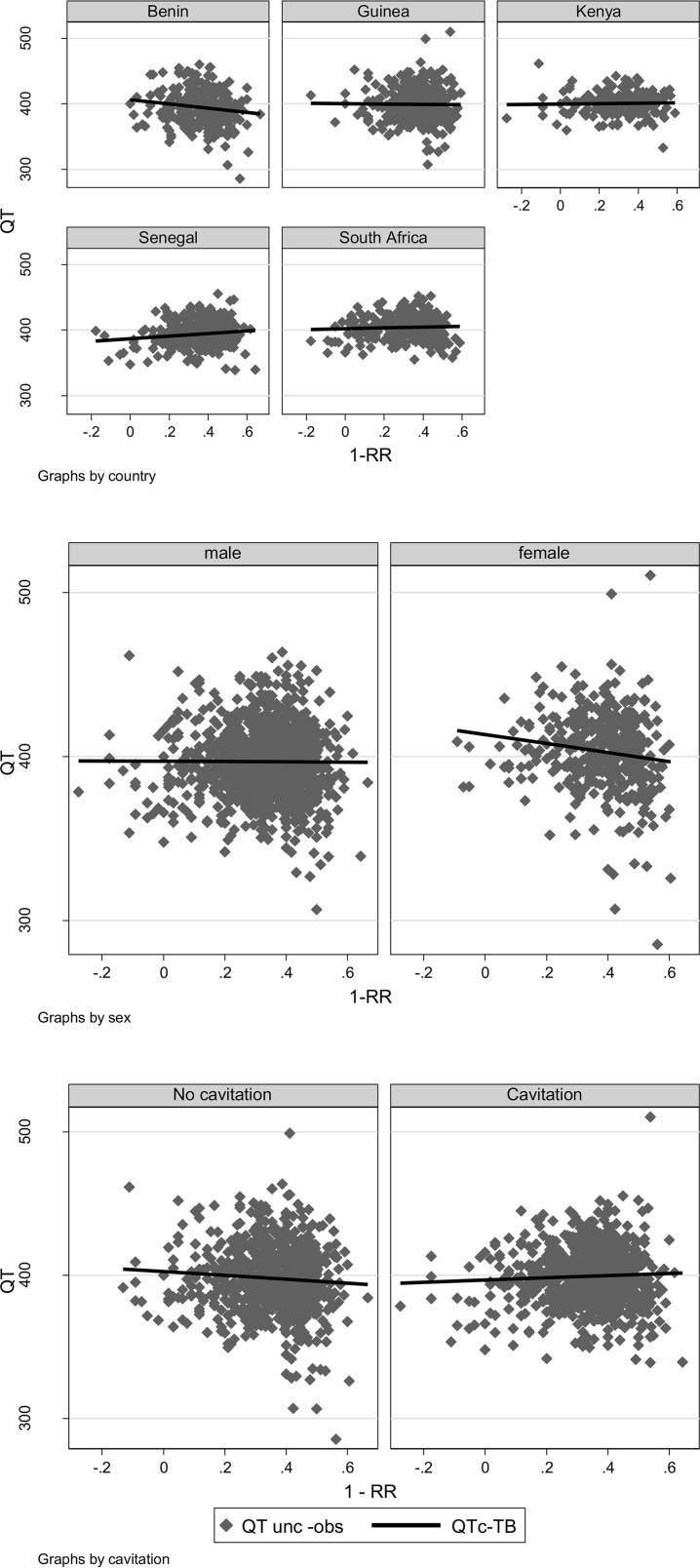
Plot of uncorrected data and QTcTB (0.4081)-corrected regression line against 1-RR (where RR = 60/heart rate), using data at baseline, by country, sex, and cavitation status.

At baseline, neither the Bazett nor the Fridericia correction was optimal; QTcB tended to undercorrect (gradient, 51.8; 95% CI, 43.5, 60.1) and QTcF tended to overcorrect (gradient, −46.3; 95% CI, −54.1, −38.5) the QT (Fig. 2; Table 3). The nonlinear model estimated the correction factor to be 0.4081 (95% CI, 0.3949, 0.4213) (QTcTB), in between the Bazett and Fridericia correction factors. This correction factor was independent of the country, sex, and presence or absence of cavitation (Fig. 3).

Applying the Bazett and Fridericia corrections and the new correction factor to QT data measured 1 and 2 months after the start of TB treatment and at the end of treatment (month 4 in the test arm and month 6 in the control arm) showed the QTcF to be a better correction, with the gradient coefficient close to zero (Table 3).

### Between-treatment comparison.

The QTcF was therefore applied for between-treatment comparisons. A total of 1,602 patients contributed to these analyses (Fig. 1). Baseline characteristics were similar between the two treatment arms (Table 1).

The peak QTcF value during follow-up was >480 ms in 21 patients overall: 0.9% (7/804) and 1.8% (14/798) in the test and control arms, respectively (Table 3). There were nine occasions of QTcF being >500 ms (Tables 3 and 4). Five occurred in the test arm (0.6%) at month 1 (506 and 514 ms), month 2 (518 ms), and month 4 (502 and 511 ms), and four occurred in the standard treatment arm (0.5%) at month 2 (510 and 517 ms) and month 6 (507 and 569 ms). The risk differences for QTcF of >480 ms and >500 were −0.9% (95% CI, −2.0% to 2.3%; *P* = 0.12) and 0.1% (95% CI, −0.6% to 0.9%; *P* = 0.75), respectively, between the test and control arms. Overall, 107 (6.7%) patients had a peak measurement change from baseline of >60 ms, with no difference between the two treatment arms (adjusted difference, 0.7%; 95% CI, −1.5% to 3.0%; *P* = 0.53).

**TABLE 4:** Comparison of on-treatment Fridericia correction QT values by study arm

Parameter and unit of measurement	Test (*n* = 804)	Control (*n* = 798)	Difference (95% CI)^*e*^	*P* value
Peak value in follow-up, mean (SD), ms	407.2 (23.3)	404.5 (25.3)	2.6 (0.2, 4.9)	0.030
Change from baseline	22.9 (26.2)	19.1 (28.1)	3.8 (1.1, 6.4)	0.005
Peak value in follow-up, % (*n*)				
>450 ms^*a*^	4.3 (35)	4.4 (35)	**0.2 (−1.6, 2.0)**	0.83
>480 ms^*b*^	0.9 (7)	1.8 (14)	**−0.9 (−2.0, 2.3%)**^*d*^	0.12
>500 ms^*c*^	0.6 (5)	0.5 (4)	**0.1 (−0.6, 0.9)**^*d*^	0.75
Change from baseline, >60 ms	7.0 (56)	6.4 (51)	**0.7 (−1.5, 3.0)**	0.53

a Timing of peak value of >450 ms: test arm, *n* = 12, 10, and 13 at month 1, month 2, and end of treatment, respectively; control arm, *n* = 15, 9, and 24 at month 1, month 2, and end of treatment, respectively.

b Timing of peak value of >480 ms: test arm, *n* = 3, 1, and 3 at month 1, month 2, and end of treatment, respectively; control arm, *n* = 5, 4, and 5 at month 1, month 2, and end of treatment, respectively.

c Timing of peak value of >500 ms: test arm, *n* = 2, 1, and 2 at month 1, month 2, and end of treatment, respectively; control arm, *n* = 0, 2, and 2 at month 1, month 2, and end of treatment, respectively.

d Not adjusted for country.

e Values shown in bold indicate risk difference (95% CI).

**FIG 4:**
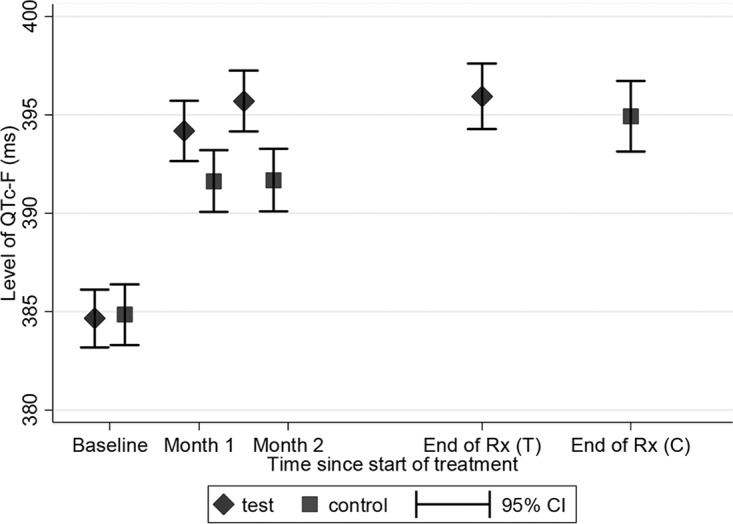
Mean and 95% confidence interval (CI), by test and control arm, of QTcF values at baseline, months 1 and 2, and end of treatment (months 4 and 6, respectively).

The overall mean peak QTc value was moderately higher in the test than in the control arm (adjusted mean difference, 2.6 ms [95% CI, 0.2, 4.9]; *P* = 0.030). The mean (95% CI) QTcF values at baseline, month 1, month 2, and end of treatment were 384.7 ms (383.2 to 386.1), 394.2 ms (392.6 to 395.7), 395.7 ms (394.1 to 397.3), and 395.9 ms (394.2 to 397.5) for the test arm and 385.1 ms (383.6 to 386.6), 391.6 ms (390.1 to 393.2), 391.7 ms (390.1 to 393.3), and 394.9 ms (393.1 to 396.7) for the control arm, respectively (Fig. 4).

### Drug levels.

Pharmacokinetic measures were available for 291 patients at month 1 (144 and 147, respectively, in the test and control arms). The *C*_max_, time to maximum concentration of drug in serum (*T*_max_), and area under the concentration-time curve (AUC) achieved by the individual drugs in the two treatment arms are summarized in Table 5. There was no evidence that *C*_max_ of any of the drugs individually was associated with QTcF at month 1 (Table 5).

**TABLE 5:** *C*_max_, *T*_max_, and AUC at steady state for each drug, by study arm (*n* = 291)

Drug and parameter at steady state	Median (minimum to maximum) for study arm:		Estimated gradient (95% CI), *P* value^*a*^
	Test (*n* = 144)	Control (*n* = 147)	
Gatifloxacin			
*C*_max_	3.8 (2.5–5.8)	NA^*b*^	−3.82 (−11.78, 4.14), 0.34
*T*_max_	1.7 (0.8–3.6)	NA	NA
Ethambutol			
*C*_max_	NA	3.2 (1.5–5.5)	−1.99 (−5.96, 1.98), 0.32
*T*_max_	NA	2.5 (1.5–4.5)	NA
Isoniazid			
*C*_max_	3.1 (0.7–8.0)	3.1 (0.5–6.0)	0.86 (−1.11, 2.83), 0.39
*T*_max_	0.9 (0.6–3.2)	0.8 (0.3–3.6)	NA
Rifampin			
*C*_max_	6.3 (1.4–13.2)	6.9 (2.0–15.6)	0.16 (−1.08, 1.39), 0.81
*T*_max_	2.2 (1.3–5.6)	1.9 (1.1–5.3)	NA
Pyrazinamide			
*C*_max_	35.9 (23.8–60.4)	35.0 (21.9–62.1)	−0.28 (−0.66, 0.10), 0.15
*T*_max_	1.7 (0.9–4.5)	1.5 (0.8–5.0)	NA

a For the association of each drug *C*_max_ individually on QTcF at month 1, adjusted for country, sex, age, and QTcF at enrollment, and study arm (only for isoniazid, rifampin, and pyrazinamide). CI confidence interval.

b NA, not applicable.

## DISCUSSION

This study shows that the risk of QT prolongation with either a 4-month regimen including gatifloxacin or a standard 6-month treatment is low: only five (0.6%) and four (0.5%) subjects, respectively, had a value of >500 ms, and 7% and 6.3%, respectively, had a prolongation relative to their baseline value of >60 ms.

We also found that in this African population with active PTB, the Bazett formula QTcB (QT/RR^0.5^) undercorrects and the Fridericia formula QTcF (QT/RR^0.33^) overcorrects QT as RR increases; the QTcTB (QT/RR^0.4081^) best fits this population. For instance, screening patients for values of >480 ms with the QTcF would have missed 1 of the 2 cases, and the QTcB would have excluded 3 more cases. While the TB correction factor appears to benefit subjects of both sexes in all the countries of this study, it will be important to verify the appropriateness of this correction on larger and more diverse TB patient populations. This may have implications for entry criteria when recruiting into a TB treatment trial, as well as measuring relative changes in the QT after treatment. As patients on treatment recover, the Fridericia formula becomes more appropriate, and QTcB and QTcTB overestimate the prolongation (with 11, 13, and 8 cases having QTcB and 2, 3, and 3 cases having QTcTB of >480 ms at week 4, week 8, and end of treatment, respectively). The correction factor is introduced to make the QT independent of the heart rate, which translates to the regression lines displayed in Fig. 2 and 3 for corrected QTc; the slope is closest to zero (a horizontal line) when using the population-specific QTcTB.

ECGs were done before starting and during antituberculosis treatment. During treatment, the ECGs were done 1 to 5 h postdosing (corresponding to the interval when drug concentrations are expected to be highest in plasma) at months 1 and 2 and at the end of treatment (month 4 for the gatifloxacin-containing regimen or month 6 for standard treatment). These measurements occurred when drug concentrations were at steady state, and patients were improving or convalescent.

It is becoming increasingly clear that, while FQs are generally known to block the inward delayed rectifier current I_Kr_ through the potassium channel, QT prolongation and TdP risk cannot be considered a class effect, as the individual FQ affinities for the hERG-I_Kr_ receptor (both in absolute terms and relative to plasma levels) vary widely.

*In vitro*, gatifloxacin had a 50% inhibitory concentration (IC_50_) of 130 μM (48.8 μg/ml) for the hERG channel I_Kr_ with blocking activities for other quinolones ranging from 18 μM (sparfloxacin) as the most active to 1,420 μM (ofloxacin) as the least active quinolone (4). A similar range of blocking activities for I_Kr_ has been determined in the mouse tumor cell line AT-1, with IC_50_s of 0.23 μM (sparfloxacin), 26.5 μM (gatifloxacin), and 27.2 μM (grepafloxacin) (8). The influence of a series of fluoroquinolones on action potential duration (APD) was also studied in isolated guinea pig right ventricular myocardia: while some of the drugs tested did not influence APD, gatifloxacin increased the APD by 13% at a concentration of 100 μM (37.5 μg/ml); at the same concentration, sparfloxacin increased the APD by 41%, while grepafloxacin and moxifloxacin showed intermediate values of 24% and 25%, respectively (9).

All the FQs tested showed propensities for a prolongation of the QT and/or the QTc interval (Carlsson correction, QT − 0.175 [RR − 300]) in an *in vivo* anesthetized rabbit model. The compounds were infused intravenously at a dose of 2 mg/kg of body weight/min for 30 min, with sparfloxacin producing the highest absolute QT prolongation (+129 ms from baseline); gatifloxacin showed a minimal prolongation of the QT interval (increase from baseline, 14 ms). Ventricular tachycardia and TdP were elicited only by sparfloxacin and only when the infusion was extended to a duration of 60 min (8). A similar model in rabbits using intravenous infusion doses of 4 mg/kg/min yielded QT and QTc interval prolongation values for gatifloxacin similar to those of sparfloxacin, with increases in interval times from about 160 ms at baseline to about 320 ms at 30 min after the start of the infusion (10).

In order to put the nonclinical data into perspective, these concentrations that evoke cardiac effects in experimental *in vitro* and *in vivo* models must be compared to plasma levels achieved in patients. According to the Tequin (gatifloxacin) label, a 400-mg intravenous bolus given to healthy volunteers leads to a *C*_max_ of ∼5.5 μg/ml, a concentration which is ∼23 times lower than the IC_50_ for hERG inhibition and ∼5-times lower than the IC_50_ for I_Kr_ blockade in AT-1 cells; in this phase 3 trial (oral treatment with 400 mg/day), the *C*_max_ was 3.9 μg/ml after the first dose and 3.8 μg/ml at steady state (IC_50_/*C*_max_ ratio, ∼34 [95% CI, 21 to 54]). Both indicate a substantially lower risk than that inferred by Kang et al. (4). In addition, when applying a scaling factor of 0.324 for the dose administered to extrapolate the *in vivo* rabbit data to humans, the intravenous infusion of 2 mg/kg/min, resulting in only a minimal prolongation of the QT interval, will then correspond to a human-equivalent bolus dose of ∼20 mg/kg, or 1,000 mg for a 50-kg human. Similarly, the FDA data for Tequin in mongrel dogs, where no influence on the ECG was seen at an intravenous infusion of 10 mg/kg/min, can be translated into a human-equivalent bolus dose of ∼162 mg/kg, or a dose of >8,000 mg. All these data suggest a low risk for gatifloxacin to induce serious cardiovascular adverse events.

Furthermore, there is no clear correlation between hERG-I_Kr_ receptor affinity and risk of QT prolongation or risk of TdP. The risk of TdP with FQs is in actual fact very low and is estimated to be at ∼27 for 10 million prescriptions for gatifloxacin, including subjects with concomitant risk factors (11). The Uppsala Monitoring Centre database reports as of 1 March 2014 a total of 13,556 cardiac adverse events with fluoroquinolones, of which 767 are QT prolongation and 451 are TdP: 100 and 53, respectively, with gatifloxacin; 207 and 166, respectively, with levofloxacin; and 269 and 113, respectively, with moxifloxacin. Direct comparisons are obviously not possible due to the absence of the denominator (number of people exposed to the different FQs).

The main methodological limitation of this study is that there was no external review of QTc measurement, but all were measured automatically with the same machine in all study sites, and all QTc values reported in the case record form (CRF) were reviewed by an external monitor; furthermore, there was only one QTc measurement done at each time point. Another potential limitation is that, assuming that *C*_max_ is the main determinant of the risk for QT prolongation, ECGs were done during treatment when all drugs were at steady state, but peak plasma concentrations might have been higher in the earlier phases of treatment.

In summary, this study indicates that neither a standard 6-month TB treatment nor a 4-month, 6-day-a-week regimen including gatifloxacin at 400 mg/day in combination with three (rifampin, isoniazid, and pyrazinamide) other antituberculosis drugs for the first 2 months and two (rifampin and isoniazid) for the following 2 months appears to carry a sizable risk of QT prolongation.

These results are significant and novel for a number of reasons. To our knowledge, this is to date the largest data set studying the QT interval during acute active tuberculosis itself, documenting the effects on the QT interval of the standard regimen as well as a fluoroquinolone-containing regimen and investigating the relationship between drug levels and the QT. As such, the data fill a knowledge gap and are useful for future studies. It will be important to verify in other sets of patients, including those with other forms of tuberculosis, whether and how active disease affects the QT interval and which formula is best suited to correct it so as to make it independent of the heart rate. This knowledge will improve also our understanding of treatment effects, as it will refine the classification of QT values as being normal or prolonged—both for eligibility for treatment and for assessing risks. This study also provides a reference point for other studies which will aim to evaluate the effects on ventricular repolarization of standard and alternative treatments on both newly diagnosed and drug-resistant tuberculosis, as the latter in particular may include drugs with potential for QT prolongation.

## MATERIALS AND METHODS

### Study design.

The study was a noninferiority randomized, open-label, controlled trial, conducted in five African countries, Benin, Guinea, Kenya, Senegal, and South Africa, with a nested PK/PD study for a subset of patients. Its objective was to assess the efficacy and safety of a gatifloxacin-containing 4-month regimen for the treatment of drug-susceptible pulmonary tuberculosis compared to standard World Health Organization (WHO)-recommended 6-month treatment (12). The protocol was approved by the relevant ethics committee and regulatory authorities of all partner institutions. More details on study design have been published elsewhere (7).

### Subjects.

Male and female patients, aged 18 to 65 years, newly diagnosed with microscopically proven pulmonary tuberculosis and providing informed consent for inclusion in the trial, were considered for enrollment. Patients with congenital QTc interval prolongation of >480 ms, clinically significant bradycardia (40 beats/minute), or hypokalemia of grade 1 and above (i.e., <3.0 meq/liter) and patients using drugs known to prolong QT interval were excluded at enrollment.

### Treatment arms.

Patients were randomized, stratified by country, to one of two treatment arms. The control treatment regimen included isoniazid (H), rifampin (R), pyrazinamide (Z), and ethambutol (E) given daily for 2 months followed by 4 months with isoniazid and rifampin (i.e., 2RHEZ/4RH). In the intervention arm (referred to as test), gatifloxacin (G) was substituted for ethambutol in the 2-month intensive phase and was maintained for the 2-month continuation phase (i.e., 2RHGZ/2GRH). Gatifloxacin was given at a dose of 400 mg per day, irrespective of body weight. The doses of HRZE followed World Health Organization (WHO) recommendations (12) and were provided as fixed-dose combination tablets.

### Measurements.

Along with clinical and laboratory evaluations, 12-lead electrocardiograms (ECGs) were performed at baseline (pretreatment), at months 1 and 2 of TB treatment, and at the end of the treatment. ECGs were obtained with a Shiller CP300 machine which was configured to report QT intervals automatically and to calculate the corrected QT interval (QTc) by Bazett's formula. The exact heart rate at the time of ECG measurement was also automatically measured and recorded. This allowed us to calculate *a posteriori* QTc interval using other formulas such as Fridericia's formula. The following information was also recorded for each patient: gender, age, medical history, vital signs (including body temperature), clinical examination, and concomitant medication.

Plasma samples were taken for drug concentration measurements at baseline and month 1 as part of a population PK study. Patients were randomized to one of three sampling schedules (A, B, and C), each with three sampling times: schedule A, sample 1, within the hour before the treatment dose (−1 to 0 h); sample 2, between 1 and 2 h after the treatment dose; and sample 3, between 2.5 and 3.5 h after the treatment dose; schedule B, sample 1, between 1 and 2 h after the treatment dose; sample 2, between 2.5 and 3.5 h after the treatment dose; and sample 3, between 4 and 6 h after the treatment dose; and schedule C, sample 1, between 1 and 2 h after the treatment dose; sample 2, between 2.5 and 3.5 h after the treatment dose; and sample 3, between 8 and 10 h after the treatment dose.

Population pharmacokinetic models were used to generate individual estimates of peak drug concentration (*C*_max_) and time to *C*_max_ (*T*_max_) at steady state (13, 14).

Drug safety was closely monitored during the course of the study in compliance with ICH/good clinical practice (GCP) guidelines.

### Statistical methods. (i) Review of the correction factor.

The QT measurement at enrollment, combined across treatment arm, was used to assess the correction factor in this sample of TB patients. We calculated the linear regression coefficients of gradient and intercept for the Bazett-corrected (correction factor RR^0.5^) and Fridericia-corrected (correction factor RR^0.33^) QT against 1-RR. These analyses were repeated for each measurement postrandomization—month 1, month 2, and at the end of treatment (either month 4 or month 6 for the test and control arms, respectively)—combined across treatment arm for the purpose of assessing the adequacy and robustness of the correction factors.

In addition, a nonlinear model was fitted to the uncorrected QT at baseline to estimate the population-specific correction factor for the pretreatment patients with active pulmonary tuberculosis.

### (ii) Definition of outcomes.

According to ICH guidelines (6), QTc data are presented as both continuous and binary variables using the Fridericia correction (QTcF). Continuous measurements were summarized using the arithmetic mean and standard deviation (SD). Peak QTc was defined as the maximum QTc interval from up to a possible 3 follow-up recordings (week 4 or 8 or end of treatment).

### (iii) Between-treatment arm comparisons.

Peak QTcF during follow-up and change of this measurement from baseline were compared between treatment arms using linear regression, adjusting for country where possible. Peak values were also classed as binary variables using cut points at >450 ms, >480 ms, and >500 ms and change from baseline as >60 ms; between-treatment comparisons were expressed as risk difference, adjusted for country. Patients with a baseline measurement and at least one follow-up measurement contributed to these analyses.

### (iv) PK/PD analysis.

In the subset of patients who had drug concentration measurements, the effect of *C*_max_, for each drug separately, on QTcF at month 1 was assessed using linear regression, adjusting for country, sex, age, and QTcF at enrollment, and study arm (adjusting for study arm only for the effect of *C*_max_ for isoniazid, rifampin, and pyrazinamide).

## References

[1] Garnett CE, Zhu H, Malik M, Fossa AA, Zhang J, Badilini F, Li J, Darpo B, Sager P, Rodriguez I 2012 Methodologies to characterize the QT/corrected QT interval in the presence of drug-induced heart rate changes or other autonomic effects. Am Heart J 163:912–930. doi:10.1016/j.ahj.2012.02.023. 22709743

[2] Owens RC Jr, Ambrose PG 2005 Antimicrobial safety: focus on fluoroquinolones. Clin Infect Dis 41(Suppl 2):S144–S157. doi:10.1086/428055. 15942881

[3] Sanguinetti MC, Jiang C, Curran ME, Keating MT 1995 A mechanistic link between an inherited and an acquired cardiac arrhythmia: HERG encodes the IKr potassium channel. Cell 81:299–307. doi:10.1016/0092-8674(95)90340-2. 7736582

[4] Kang J, Wang L, Chen XL, Triggle DJ, Rampe D 2001 Interactions of a series of fluoroquinolone antibacterial drugs with the human cardiac K+ channel HERG. Mol Pharmacol 59:122–126. 11125032 10.1124/mol.59.1.122

[5] Center for Drug Evaluation and Research. 2012 Guidance for industry. E14 clinical evaluation of QT/QTc interval prolongation and proarrhythmic potential for non-antiarrhythmic drugs: questions and answers (R1). Center for Drug Evaluation and Research, US Department of Health and Human Services, Silver Spring, MD.

[6] Department of Health and Human Services, Food and Drug Administration. 2005 International Conference on Harmonisation; guidance on E14 clinical evaluation of QT/QTc interval prolongation and proarrhythmic potential for non-antiarrhythmic drugs; availability. Fed Regist 70:61134–61135. 16237860

[7] Merle CS, Fielding K, Sow OB, Gninafon M, Lo MB, Mthiyane T, Odhiambo J, Amukoye E, Bah B, Kassa F, N′Diaye A, Rustomjee R, Dejong BC, Horton J, Perronne C, Sismanidis C, Lapujade O, Olliaro P, Lienhardt C 2014 2014. A four-month gatifloxacin-containing regimen for treating tuberculosis. N Engl J Med 371:1588–1598. doi:10.1056/NEJMoa1315817. 25337748

[8] Anderson ME, Mazur A, Yang T, Roden DM 2001 Potassium current antagonist properties and proarrhythmic consequences of quinolone antibiotics. J Pharmacol Exp Ther 296:806–810. 11181910

[9] Hagiwara T, Satoh S, Kasai Y, Takasuna K 2001 A comparative study of the fluoroquinolone antibacterial agents on the action potential duration in guinea pig ventricular myocardia. Jpn J Pharmacol 87:231–234. doi:10.1254/jjp.87.231. 11885973

[10] Akita M, Shibazaki Y, Izumi M, Hiratsuka K, Sakai T, Kurosawa T, Shindo Y 2004 Comparative assessment of prurifloxacin, sparfloxacin, gatifloxacin and levofloxacin in the rabbit model of proarrhythmia. J Toxicol Sci 29:63–71. doi:10.2131/jts.29.63. 15018156

[11] Murphy ME, Singh KP, Laurenzi M, Brown M, Gillespie SH 2012 Managing malaria in tuberculosis patients on fluoroquinolone-containing regimens: assessing the risk of QT prolongation. Int J Tuberc Lung Dis 16:144–149. doi:10.5588/ijtld.11.0074. 22236913

[12] World Health Organization. 2004 Treatment of tuberculosis guidelines for national programmes. WHO/CDS/TB/2003.313. World Health Organization, Geneva, Switzerland.

[13] Smythe WA 2016 Characterizing population pharmacokinetic/pharmacodynamic relationships in pulmonary tuberculosis infected adults using nonlinear mixed effects modelling. PhD thesis. University of Cape Town, Cape Town, South Africa.

[14] Smythe W, Merle CS, Rustomjee R, Gninafon M, Lo MB, Bah-Sow O, Olliaro PL, Lienhardt C, Horton J, Smith P, McIlleron H, Simonsson US 2013 Evaluation of initial and steady-state gatifloxacin pharmacokinetics and dose in pulmonary tuberculosis patients by using Monte Carlo simulations. Antimicrob Agents Chemother 57:4164–4171. doi:10.1128/AAC.00479-13. 23774436 PMC3754315

